# First-line surgery versus first-line assisted reproductive technology for women with deep infiltrating endometriosis: a systematic review and meta-analysis

**DOI:** 10.3389/fendo.2024.1352770

**Published:** 2024-04-18

**Authors:** Ying Liang, Min Liu, Jianmei Zhang, Zenghui Mao

**Affiliations:** ^1^ Reproductive Center of Changsha Hospital for Maternal and Child Health Care, Changsha, China; ^2^ Hunan Key Laboratory of Regional Hereditary Birth Defects Prevention and Control, Changsha Hospital for Maternal Child Health Care Affiliated to Hunan Normal University, Changsha, China

**Keywords:** assisted reproductive technology, endometriosis, meta-analysis, systematic review, fertility

## Abstract

**Background:**

The efficiency of different first-line treatments, such as first-line surgery and assisted reproductive technology (ART), in women with deep infiltrating endometriosis (DIE) is still unclear due to a lack of direct comparative trials. This systematic review and meta-analysis aim to elucidate and compare the efficacies of first-line treatments in patients with DIE, with an emphasis on fertility outcomes.

**Methods:**

An exhaustive search of PubMed Central, SCOPUS, EMBASE, MEDLINE, Cochrane trial registry, Google Scholar, and Clinicaltrials.gov databases was done to identify studies directly comparing first-line surgery and assisted reproductive technology (ART) for DIE, and reporting fertility-related outcomes. Pooled estimates for each of the binary outcomes were reported as odds ratios (ORs) with 95% confidence intervals (CIs). The results were pooled using a random-effects model with the Mantel-Haenszel technique.

**Results:**

Our results show that pregnancy rate per patient (OR, 1.47; 95% CI, 0.59 to 3.63), pregnancy rate per cycle (OR, 1.16; 95% CI, 0.45 to 2.99), and live births per patient (OR, 1.66; 95% CI, 0.56 to 4.91) were comparable in DIE patients, treated with surgery or ART as a first line of treatment. When both complete and incomplete surgical DIE excision procedures were taken into account, surgery was associated with a significant enhancement in the pregnancy rate per patient (OR, 1.63; 95% CI, 1.11 to 2.40).

**Conclusion:**

The available evidence suggests that both first-line surgery and ART can be effective DIE treatments with similar fertility outcomes. However, further analysis reveals that excluding studies involving endometriomas significantly alters the understanding of treatment efficacy between surgery and ART for DIE-associated infertility.

**Systematic Review Registration:**

https://www.crd.york.ac.uk/PROSPERO/display_record.php?RecordID=426061, identifier CRD42023426061.

## Introduction

Endometriosis, a chronic gynecological condition characterized by endometrial-like tissue growth outside of the uterus, is a major health concern affecting 6–10% of women in their reproductive years (1). Deep infiltrating endometriosis (DIE) is the most severe and debilitating form of this heterogeneous disease (2) and it frequently results in severe pain, infertility, and significantly reduced quality of life. Endometriotic lesions in DEI penetrate more than 5 mm under the peritoneum and often infiltrate vital structures such as the bowel, bladder, ureters, and in some cases, even the sciatic nerve (3).

Management of DIE remains a clinical challenge due to its multifactorial etiology and variable symptomatology (2), and aims to alleviate pain, improve fertility outcomes when desired, and enhance overall quality of life. Currently, therapeutic options include primarily surgical management and medical treatment using assisted reproductive technologies (ARTs) (2, 4).

Surgery, which can be conservative (aiming to preserve fertility) or radical (hysterectomy with or without removal of the ovaries), has long been the gold standard for managing endometriosis and particularly DIE (5). Surgical treatment aims to remove all visible endometriotic lesions to restore normal pelvic anatomy, provide symptomatic relief, and improve fertility. However, as an invasive method, surgical excision is associated with higher risk of post-surgery complications and disease recurrences (6).

By contrast, non-surgical management, consisting primarily of hormonal therapy, aims to suppress the growth of endometriotic lesions and provide symptomatic relief. ARTs, including *in vitro* fertilization (IVF), are common for managing the infertility associated with all forms of endometriosis (7). In theory, ARTs do not require anatomical corrections of endometriosis-caused alterations, and they can overcome the diminished ovarian reserve and poor oocyte quality associated with the disease (8).

However, up to date no trials attempted to directly compare first-line surgery and ARTs, and current evidence is mostly based on observational data, which can be influenced by selection biases and confounding factors. Therefore, there is still no consensus on the most effective first-line treatment for DIE, especially in terms of fertility outcomes.

This systematic review and meta-analysis aim to directly compare the effectiveness of first-line surgery versus ARTs for DIE management to provide a comprehensive analysis of the best available evidence. Our results may assist clinicians and patients in informed decision-making. Identifying the most effective fertility treatment may allow to implement individualized patient-centered care strategies and to inform future guidelines and consensus statements, contributing to the standardization of care and ultimately improving the outcomes and quality of life for women with DIE.

## Methods

### Eligibility criteria

We included eligible randomized controlled trials (RCTs) either in parallel or cluster form, and studies with both prospective and retrospective observational design. Case reports/series and unpublished grey literature were excluded from the analysis.

Protocol registration: PROSPERO, CRD42023426061.

Population: Eligible studies were conducted with women undergoing treatment for DIE.

Intervention and Comparator groups: All studies, directly comparing first-line surgery with first-line ARTs for DIE management, were considered for inclusion.

Outcomes: The primary outcomes included cumulative pregnancy rates per patient and cycle, and live birth rates per patient.

### Search strategy

Systematic search for relevant papers was carried out in multiple databases, including PubMed Central, SCOPUS, EMBASE, MEDLINE, Cochrane trial registry, Google Scholar, and Clinicaltrials.gov using medical subject headings (MeSH) and free-text terms. Appropriate Boolean operators (“AND,” “OR,” and “NOT”) were used to combine predefined search terms. The search period started from January 1964 (or database inception, whichever was earlier) to April 2023, without any language restrictions.

### Study selection

A pair of independent researchers conducted the initial study selection by examining titles, keywords, and abstracts. Both investigators obtained full-text studies for a second phase screening according to the eligibility criteria. Next, full-texts of relevant studies were read, and studies that met the eligibility criteria were selected for further analysis. We followed the “Preferred Reporting Items for Systematic Reviews and Meta-Analyses (PRISMA) checklist 2020” to write our review (9).

### Extracted data and study quality

Both researchers manually extracted data from the chosen full-text articles using predefined semi-structured data collection forms. Quality of the included studies was evaluated using the Newcastle Ottawa (NO) scale for assessing observational studies (10) on the basis of selection, comparability, and outcome domains, and each study was then classified as having good or poor quality.

### Statistical analysis

All the outcomes in our analysis were binary. Number of events and participants in each group was entered and analyzed. Pooled estimates were reported as odds ratios with 95% confidence intervals (CIs). Random-effects model with Mantel-Haenszel technique was used to pool the results to account for the potential clinical and methodological heterogeneity (11). Forest plots were constructed to visually inspect estimates, and the heterogeneity was assessed by the *I^2^* statistic and Chi-squared test (11). We were unable to assess publication bias and perform meta-regression due to insufficient number of studies (less than 10).

To enhance the robustness of our findings and address potential sources of heterogeneity, additional sensitivity analyses were performed by excluding studies that significantly influenced the pooled estimates. Specifically, the study by Maignien et al. (2020) (12) was identified as a potential outlier contributing to statistical heterogeneity. Its exclusion aimed to assess the stability of our results and the potential impact of individual studies on the overall conclusions. This approach allows us to provide a more nuanced interpretation of the data, acknowledging the diverse methodologies and populations represented in the literature. All statistical analyses were done using STATA version 14.2.

## Results

**Figure 1:**
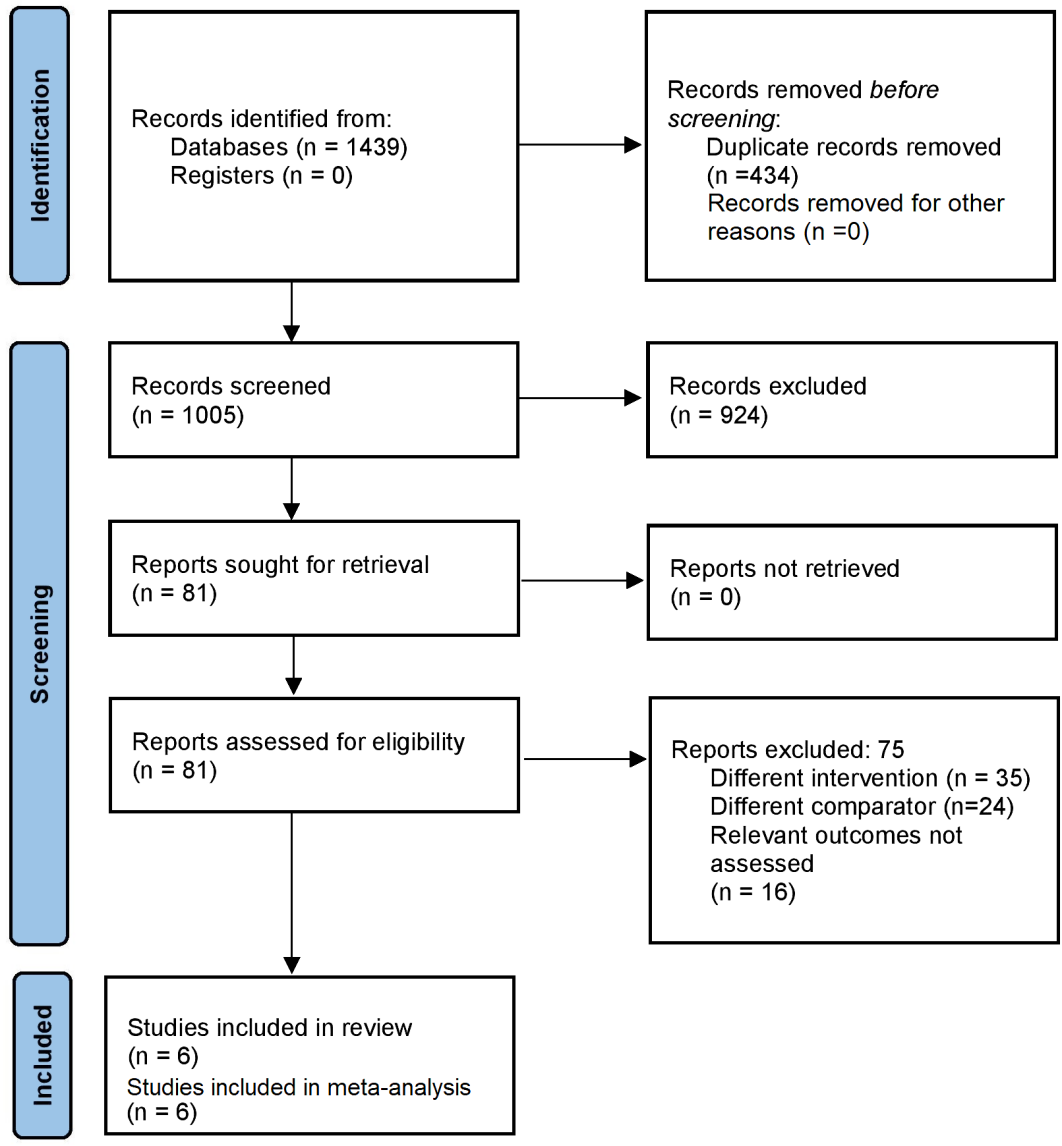
Search Strategy.

After the primary screening, 1439 citations were identified across the databases. Following duplicates removal, 81 full-text articles were retrieved for the secondary screening. Finally, 6 studies that satisfied our eligibility criteria were selected for the analysis (**Figure 1**) (12–17).

All studies except one (Bianchi 2009 (14), a Brazilian prospective cohort study, were retrospective and conducted in France. Sample sizes in the included studies varied between 72 and 222. Most studies, except for the one by Rubod 2019 (15), had patient’s preferences as allocation criteria. Mean ages of the participants ranged from 31 to 33 years. Overall, 4 out of 6 included studies had high risks of bias (**Table 1**).

**Table 1:** Details of included studies.

References	Character	Country	Size	Mean age (in years)	Allocation criteria	Participants	Surgical procedure details	Risk of bias
Bendifallah 2017 (13)	Retrospective cohort	France	Surgery: 55 ART: 55	Surgery: 31.3 ART: 32	Patients’ preferences	Women with infertility with DIE (*in situ* colorectal endometriosis)	Shaving, disc excision, or segmental resection	Low
Bianchi 2009 (14)	Prospective cohort	Brazil	Surgery: 64 ART: 105	Surgery: 32 ART: 32	Patients’ preferences	Women with infertility with clinical and TVS diagnosis of DIE (with or without colorectal endometriosis) and <38 years	Extensive laparoscopic excision of all DIE lesions and endometriomas	High
Ferrier 2023 (16)	Retrospective cohort	France	Surgery: 92 ART: 92	Surgery: 32.7 ART: 32	Patients’ preferences	Patients between 18 and 43 years with infertility associated with DIE without colorectal involvement	Complete removal of endometriotic lesions by laparoscopy	High
Maignien 2020 (12)	Retrospective cohort	France	Surgery: 155 ART: 67	All patients: 33	Patients’ preferences	DE patients who underwent *in vitro* fertilization (IVF) or intra-cytoplasmic sperm injection (ICSI) treatment	NR	Moderate
Mounsambote 2017 (17)	Retrospective cohort	France	Surgery: 35 ART: 37	Surgery: 32.1 ART: 33.1	Patients’ preferences	Women with infertility with DIE and without digestive involvement	Complete resection of endometriosis	High
Rubod 2019 (15)	Retrospective cohort	France	Surgery: 78 ART: 64	All patients: 31.1	Complete surgery: patients who were symptomatic. Incomplete surgery: asymptomatic patients only to facilitate IVF conditions. No surgery: asymptomatic patients with no affectation of IVF conditions	Women with infertility with posterior deep endometriosis	Complete surgery of all DIE lesions and endometriomas after failure of medical treatment	High

DIE, Deep infiltrating endometriosis; ART, Assisted Reproductive Technologies; TVS, Transvaginal sonography; IVF, In-vitro fertilization; NR, Not reported.

### Pregnancy rates per patient

**Figure 2:**
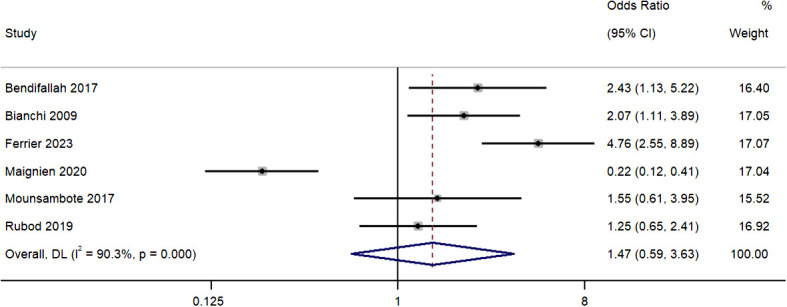
Meta-analysis in pregnancy rates per patient between first line surgery and first line ARTs. Diamond shape – pooled estimate with 95%CI; red dotted vertical line – pooled estimate.

All six studies provided information on the pregnancy rate disparities per patient between the group of patients who received surgery, and a group who received ART as the first line of treatment. Pooled OR was 1.47 (95% CI, 0.59 to 3.63; *I^2^ = *90.3%), indicating similar pregnancy rates per patient for the two groups (*P*=0.41) (**Figure 2**). The sensitivity analysis revealed that removal of the study by Maignien et al. (2020) (12) significantly altered pooled estimate to 2.21 (95% CI, 1.36 to 3.56; p<0.01) (**Supplementary Figure 1**) and contributed to statistical heterogeneity.

### Pregnancy rate per cycle

**Figure 3:**
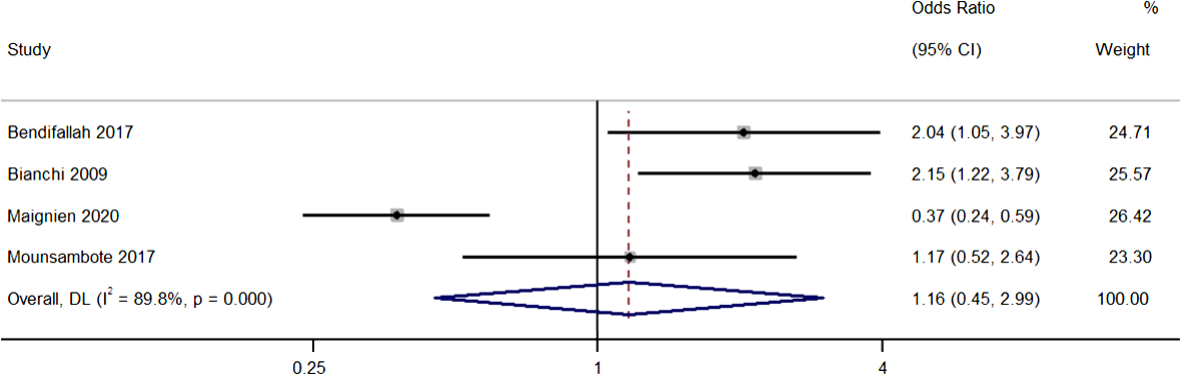
Meta-analysis in pregnancy rates per cycle between first line surgery and first line ARTs. Diamond shape – pooled estimate with 95%CI; red dotted vertical line – pooled estimate.

Four studies provided information on the differences in pregnancy rates per cycle between both group with the pooled OR of 1.16 (95% CI, 0.45 to 2.99; *I^2^ = *89.8%), indicating similar rates in the two groups (*P*=0.76) (**Figure 3**). The sensitivity analysis revealed that removal of the study by Maignien et al. (2020) (12) significantly altered pooled estimate to 1.85 (95% CI, 1.26 to 2.71; p<0.01), and contributed to statistical heterogeneity, indicating significant single study effects (**Supplementary Figure 2**).

### Live births per patient

**Figure 4:**
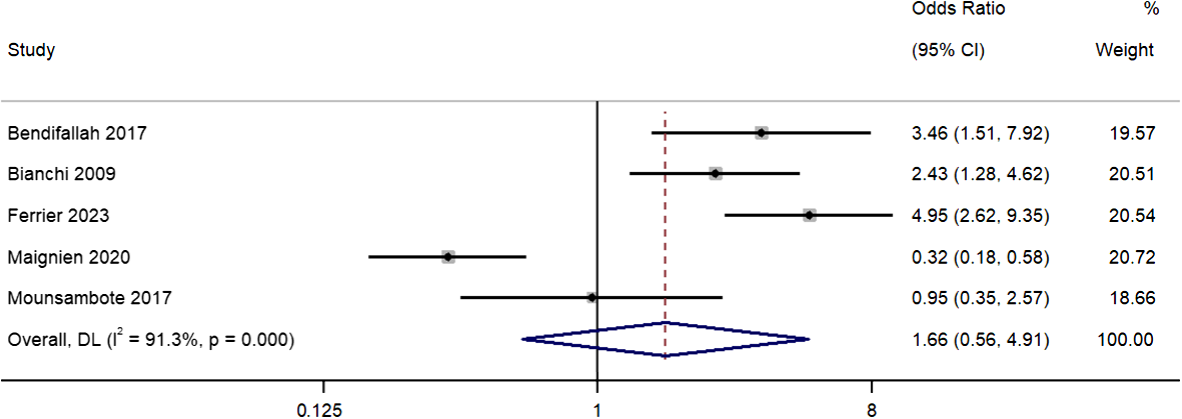
Meta-analysis in live births per patient between first line surgery and first line ARTs. Diamond shape – pooled estimate with 95%CI; red dotted vertical line – pooled estimate.

Five studies reported differences in live births per patient between the two treatment groups. The pooled OR was 1.66 (95% CI, 0.56 to 4.91; *I^2^* = 91.3%), indicating similar rates in both groups (*P*=0.36) (**Figure 4**). Our sensitivity analysis revealed that removal of the study by Maignien et al. (2020) (12) study significantly altered pooled estimate to 2.90 (95% CI, 2.01 to 4.20; p<0.01) and contributed to statistical heterogeneity, indicating significant single study effects (**Supplementary Figure 3**).

### Pregnancy rate per patient (including complete and incomplete surgery)

**Figure 5:**
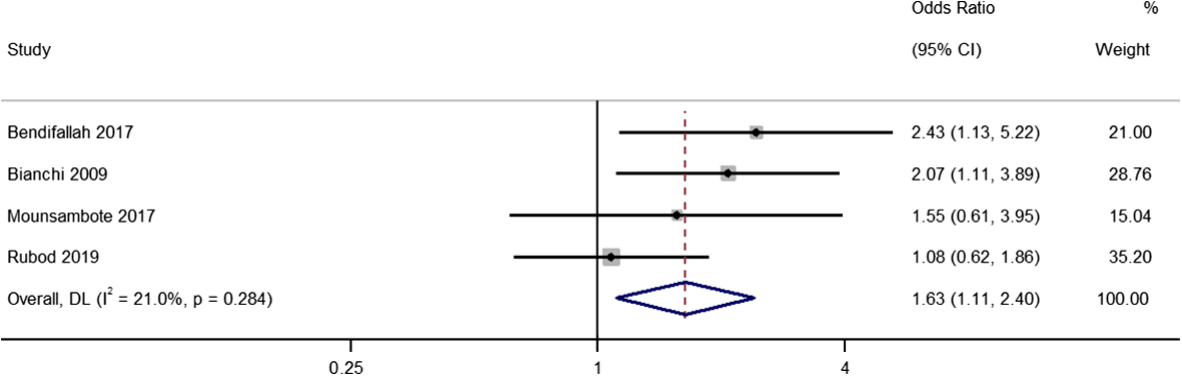
Meta-analysis in pregnancy rates per patient between first line surgery (after both complete and incomplete surgeries) and first line ARTs. Diamond shape – pooled estimate with 95%CI; red dotted vertical line – pooled estimate.

Four studies provided data on the differences in pregnancy rates per patient (including patients who underwent complete and incomplete excision) between the two treatment groups, with pooled OR of 1.63 (95% CI, 1.11 to 2.40; *I^2^* = 21%), indicating a difference between the two groups (*P*=0.01) (**Figure 5**). The sensitivity analysis did not reveal any relevant single study effects (**Supplementary Figure 4**).

### Publication bias assessment

**Supplementary Figure 5** shows the funnel plot for publication bias assessment for all the outcomes. All the plots were slightly asymmetrical indicating the possibility of publication bias.

## Discussion

DIE presents significant medical challenges due to its complex nature and often debilitating symptoms. Our study did not find significant differences in pregnancy rates per patient, pregnancy rates per cycle, and live births per patient between patients who received first-line surgery or ARTs for DIE. Our results are in contrast to the outcomes of other meta-analyses that have found significant differences favoring one treatment modality over the other (18).

This discrepancy could be attributed to the inclusion of a study by Maignien et al. (2020) (12), which has not been considered in other meta-analyses. The study by Maignien et al. significantly contributed to our pooled estimates and appeared to be a major source of statistical heterogeneity. The relevant sensitivity analysis showed that removing this study from our meta-analysis altered the pooled estimates substantially and reduced heterogeneity, aligning our findings more closely with those of other meta-analyses.

Our results demonstrate the potential significant impact of one individual study on the pooled results, especially in case of the meta-analysis with small number of included studies, and further emphasizes the need to continuously update meta-analyses as the new research emerges. Our study provides an update to previous meta-analyses, reflecting the most recent evidence in the field. However, it also underscores the need for further high-quality studies comparing first-line surgery and ARTs in patients with DIE to confirm our findings and provide more robust evidence base for clinical decision-making.

In light of the sensitivity analyses conducted, particularly following the exclusion of the study by Maignien et al. (2020) (12), our findings present a compelling shift in the pooled estimates that underscore the potential superiority of surgery over direct ART in enhancing pregnancy rates per patient and per cycle, and live births per patient among patients with DIE. This significant alteration in the landscape of our results suggests a substantial effect of surgical intervention on pregnancy outcomes, which merits a thorough examination within the context of DIE treatment paradigms.

The implications of these revised findings are multifaceted. Firstly, they signal a possible paradigm shift in the management of DIE-associated infertility, advocating for a consideration of surgical treatment as a viable first-line intervention. This is particularly relevant in scenarios where direct ART might have been previously considered the more favorable option. The strength of the evidence favoring surgery, highlighted by the new statistical significance, suggests that, in certain contexts, surgery could offer better odds for achieving pregnancy compared to direct ART interventions.

The revised analysis advocates for a patient-centered approach, considering individual patient profiles, the extent of disease, and personal fertility goals. This approach aligns with the growing body of evidence suggesting that the choice between surgery and ART should not be binary but tailored to the specific needs and circumstances of each patient. This analysis providing a balanced perspective that accounts for the complexity of treatment decisions in this context. It also sets a precedent for future research to explore the conditional benefits of surgery over ART, particularly in well-defined patient subsets, to further refine our understanding of optimal treatment pathways for DIE-associated infertility.

Importantly, our analysis revealed a high level of heterogeneity among the included studies. This heterogeneity reflects not only methodological and study design differences but also clinical and phenotypic diversity of DIE. Recent research (19) show that DIE can manifest in various anatomical locations, each with its unique impact on symptoms, treatment responses, and fertility outcomes. This variability in DIE localizations, ranging from superficial peritoneal lesions to deep infiltrating lesions involving the bowel, bladder, and other pelvic organs, contributes significantly to the clinical complexity and treatment challenges associated with this condition.

The diversity in DIE presentations necessitates a tailored approach to both surgical and ART interventions. The effectiveness and potential complications of these treatments can vary widely depending on the lesion’s location and extent. For instance, surgical removal of bowel lesions may have different implications for fertility and symptom relief compared to surgery for lesions in the ovaries or fallopian tubes. Similarly, the success rates of ARTs might be influenced by the specific DIE phenotype, with certain localizations posing more significant barriers to natural conception than others.

Acknowledging this heterogeneity is crucial for interpreting the findings of our meta-analysis and understanding the broader context of DIE management. It underscores the need for individualized treatment plans that consider the specific characteristics and preferences of each patient. Furthermore, this heterogeneity highlights the importance of advancing research that stratifies patients according to DIE localization and severity to provide more granular insights into the comparative effectiveness of first-line surgery and ARTs.

We believe the difference between our results and previous studies may also be attributed to the primary objective of surgical treatment in endometriosis, which is the removal of endometriotic lesions and restoration of normal pelvic anatomy (actions that can potentially enhance fertility by improving the physiological environment for conception) (20). ART, such as *in vitro* fertilization (IVF), bypass anatomical challenges associated with endometriosis but does not directly address the underlying pathology (21). In addition, ART, particularly IVF, has shown promising results in the management of infertility associated with endometriosis (21). By bypassing the distorted anatomical features and facilitating the fertilization process in a controlled environment, ARTs can effectively overcome some of the fertility challenges posed by endometriosis. However, the success of ART is influenced by diverse factors, including woman’s age, ovarian reserve, and the quality of embryos (22). Thus, while ARTs may be a valuable tool for infertility management in women with DIE, its effectiveness as a first-line treatment needs further exploration.

The choice between first-line surgery and ART difficult and medical practitioners should consider various factors, including patient’s age, the severity of symptoms, the desire to achieve pregnancy, and individual risk factors (23). For example, younger patients with more severe symptoms may benefit more from surgery, while older patients may prefer ART to improve the immediate fertility outcomes (24).

Finally, the effectiveness of surgical interventions may depend on the extent and location of the endometriotic lesions. For example, deep infiltrating lesions involving vital structures like the bowel or bladder may require more complex surgical procedures, potentially increasing the risk of complications (24). Additionally, the skill and experience of the surgeon play a significant role in the success of the procedure and the subsequent fertility outcomes. All these factors may account for some of the variability in the outcomes of surgical management in the included studies.

Our study showed that surgery, whether complete or incomplete, resulted in improved pregnancy rates per patient. This finding underscores the importance of surgical interventions as a DIE treatment: even if surgery does not completely eradicate the disease, it may still provide substantial benefits in terms of fertility outcomes. However, the choice of surgery should always be balanced with its potential risks, and these should be thoroughly discussed with patients.

The effect of complete versus incomplete surgery in the management of DIE is another important consideration. During incomplete surgery, some endometriotic lesions are left behind due to the risk of damage to vital structures. This may provide temporary relief of symptoms, but leaves patients with high recurrence risks (25). Complete excision aims for total eradication of endometriotic tissue, potentially providing long-lasting relief and improved fertility outcomes. However, it carries a higher risk of complications than the incomplete procedure and requires a skilled surgeon with experience in DIE management.

Therefore, there is a need for a multidisciplinary approach for DIE management that involves gynecologists, pain specialists, psychologists, and fertility specialists to provide comprehensive care addressing all aspects of the disease.

While the results of our analysis provide valuable insights, we should acknowledge certain limitations. High level of heterogeneity among the included studies, particularly in the definition of outcomes, study populations, and treatment protocols, may have influenced our findings. And, we found a high risk of bias in some included studies, which could affect the reliability of our pooled estimates. This indicates that the overall strength of evidence in this analysis is low, reflecting heterogeneity among studies, variable treatment protocols and inclusion of observational studies. This necessitates cautious interpretation of our findings and underscores the need for further, high-quality research to validate these preliminary outcomes and enhance the evidence base in the field of DIE management. Due to the limited number of studies included in our meta-analysis, we were unable to perform funnel plot asymmetry evaluations, such as the Egger or Begg test, to assess for potential publication bias as recommended for analyses with 10 or more studies (11).

We believe that our results will contribute to the ongoing discussion on the optimal first-line treatments for DIE (26). Given the complexity and heterogeneity of the disease, it is unlikely that a one-size-fits-all approach will be effective. Instead, individualized treatment plans, considering patient’s specific circumstances and preferences, should be the goal. Currently ongoing multicentric trials may provide deeper understanding of the comparative effectiveness of the different treatment options, including surgery and ARTs (27).

In light of emerging evidence on the impact of endometriosis on reproductive outcomes, it is crucial to consider the severity of the condition when evaluating treatment efficacy and pregnancy success. Numerous studies showed that severe endometriosis, particularly forms that deeply infiltrate pelvic structures, is associated with adverse outcomes in placentation and overall pregnancy outcomes (28, 29). These findings underscore the importance of precise classification systems that account for the severity and specific characteristics of endometriosis in research and clinical practice. In this context, the Enzian classification (30) emerges as a valuable tool for future studies aimed at dissecting the nuanced relationship between endometriosis severity and reproductive outcomes. This classification system provides a detailed framework for categorizing the extent and location of deep endometriosis, and can facilitate more nuanced analyses and comparisons across studies. Applying the Enzian classification in future research could enhance our understanding of how varying severities of deep endometriosis impact fertility and pregnancy, enabling clinicians to tailor treatment strategies more effectively to individual patient profiles and ensure more personalized approaches to treatment. It could also help in developing predictive models for fertility outcomes in patients with endometriosis, thereby improving patient counselling and management decisions. Nonetheless, it is important to note that any woman with endometriosis requires either no surgery or only one surgery in their entire lifetime and hence, the timing of this surgery matters the most.

In conclusion, while both first-line surgery and ART can be effective DIE treatments with similar fertility outcomes, our results highlight the need for further well-designed, direct comparative trials between first-line surgery and ARTs in DIE management. Our comprehensive sensitivity analysis, specifically the exclusion of studies with endometriomas, has markedly shifted the landscape of our findings, emphasizing the potential of surgical approaches in enhancing fertility outcomes over direct ART in certain contexts of DIE. It highlights the critical importance of individualized patient assessments, considering the presence and impact of endometriomas on fertility and treatment efficacy. Consequently, our study advocates for a more stratified approach to treatment decision-making, ensuring that patients receive the most suitable intervention based on their unique clinical profiles. Ultimately, these insights pave the way for further research into the differential impacts of endometriosis subtypes on reproductive outcomes, aiming to refine treatment protocols and improve prognoses for women battling this multifaceted condition.

## Data availability statement

The datasets presented in this study can be found in online repositories. The names of the repository/repositories and accession number(s) can be found in the article/**Supplementary Material**.

## Author contributions

YL: Conceptualization, Investigation, Methodology, Writing – original draft. ML: Conceptualization, Investigation, Methodology, Project administration, Resources, Writing – original draft. JZ: Conceptualization, Formal analysis, Investigation, Methodology, Validation, Writing – original draft. ZM: Conceptualization, Data curation, Investigation, Methodology, Validation, Writing – review & editing.
