# Supplementary figures and images for: First-line surgery versus first-line assisted reproductive technology for women with deep infiltrating endometriosis: a systematic review and meta-analysis
Source: Front Endocrinol (Lausanne). 2024 Apr 18;15:1352770. doi: 10.3389/fendo.2024.1352770 (PMC11063350; doi:10.3389/fendo.2024.1352770)

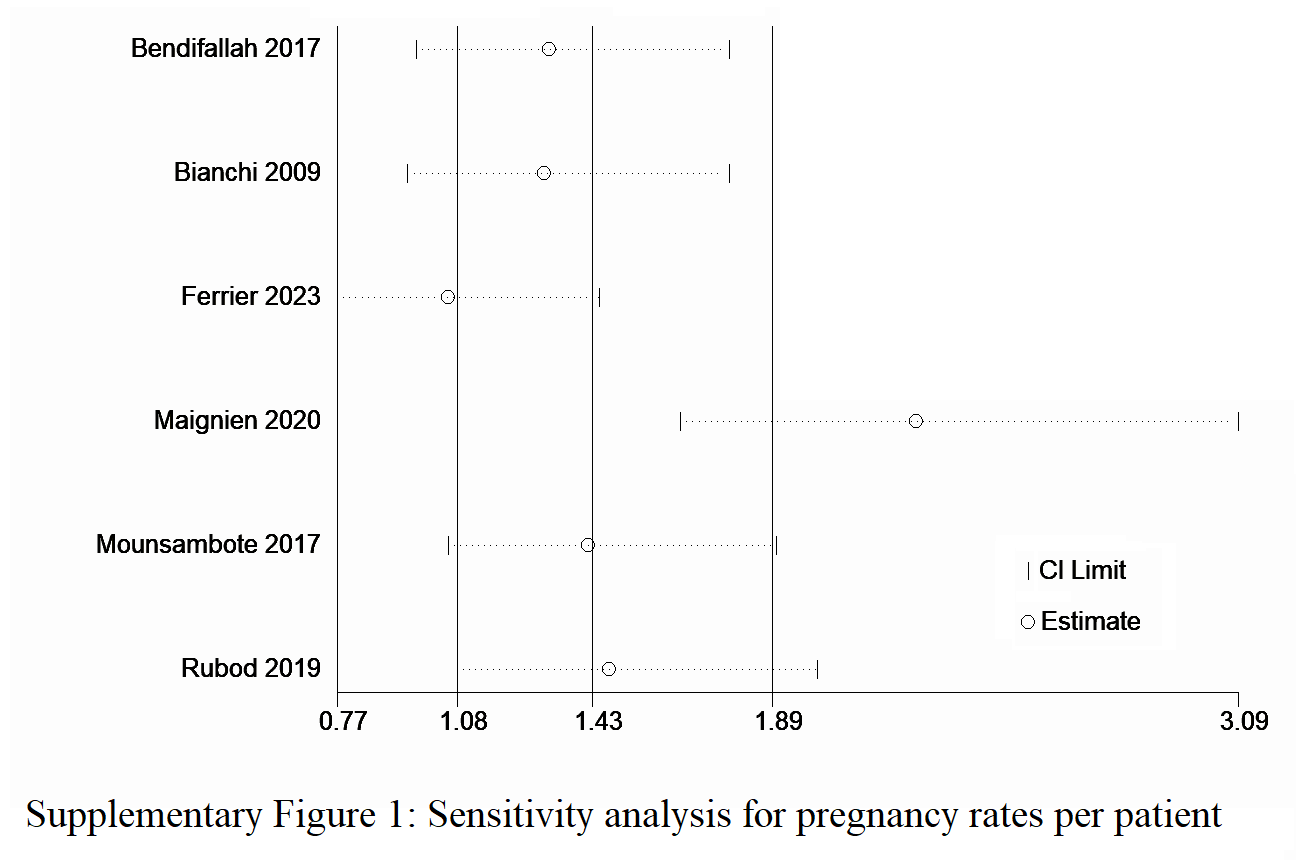

Supplement: Supplementary file 1 [file Image_1.tif]

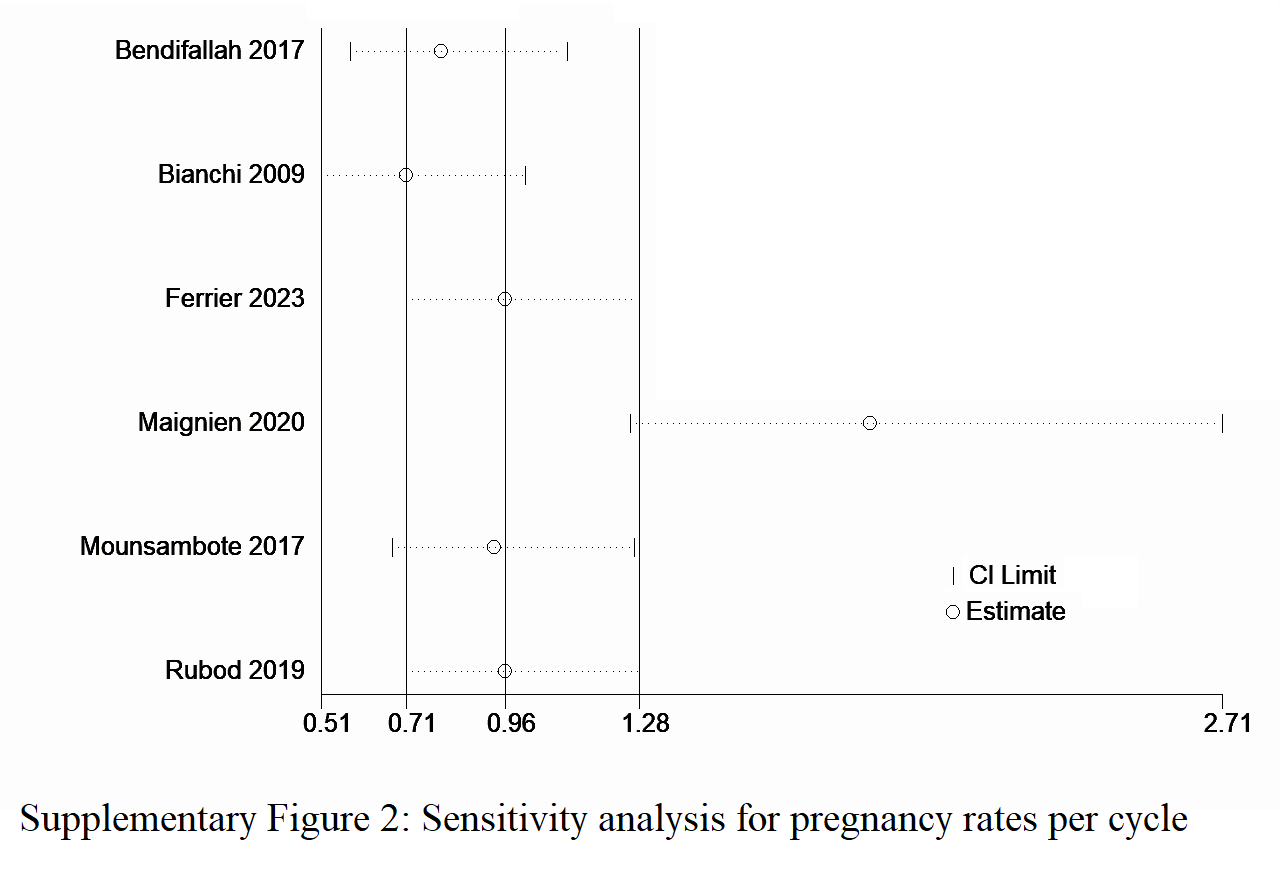

Supplement: Supplementary file 2 [file Image_2.tif]

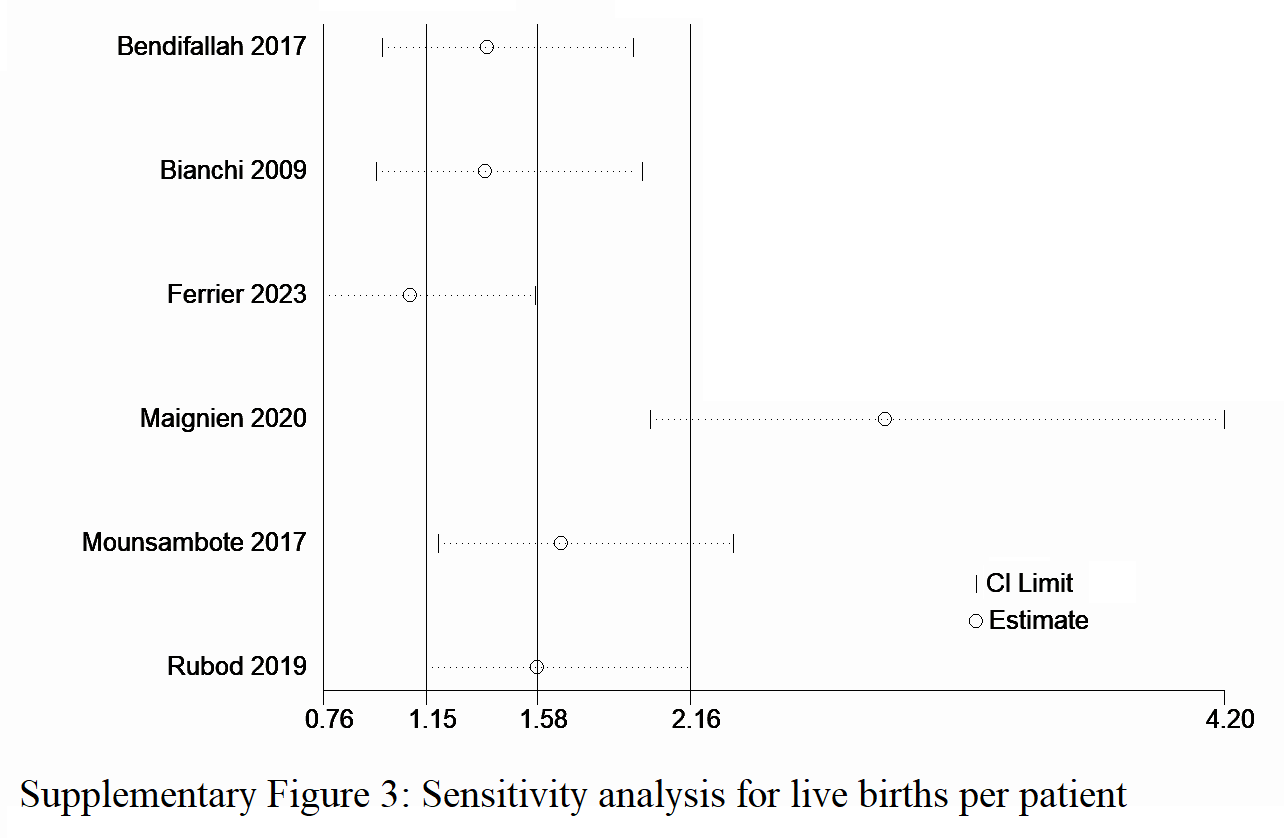

Supplement: Supplementary file 3 [file Image_3.tif]

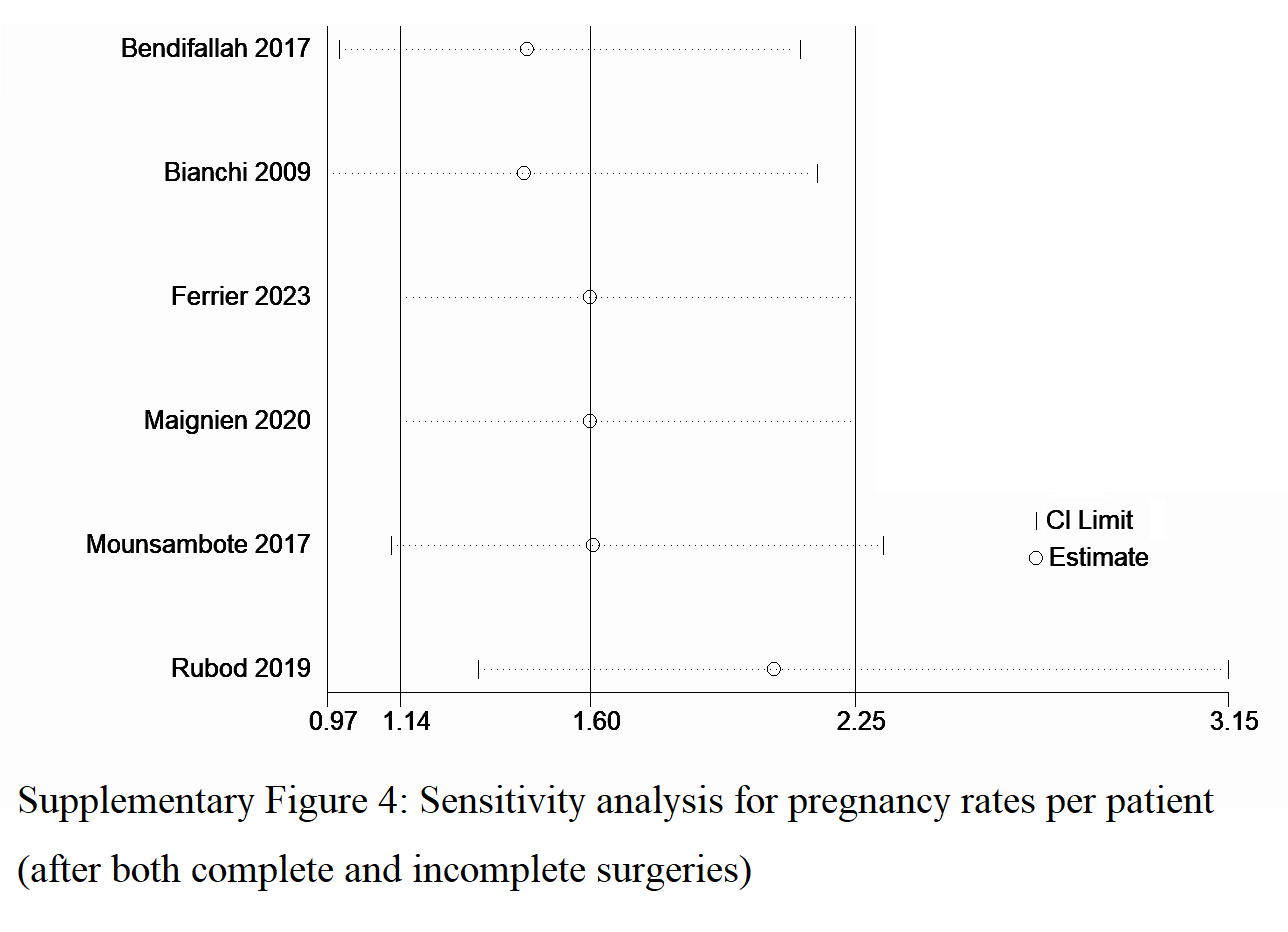

Supplement: Supplementary file 4 [file Image_4.tif]

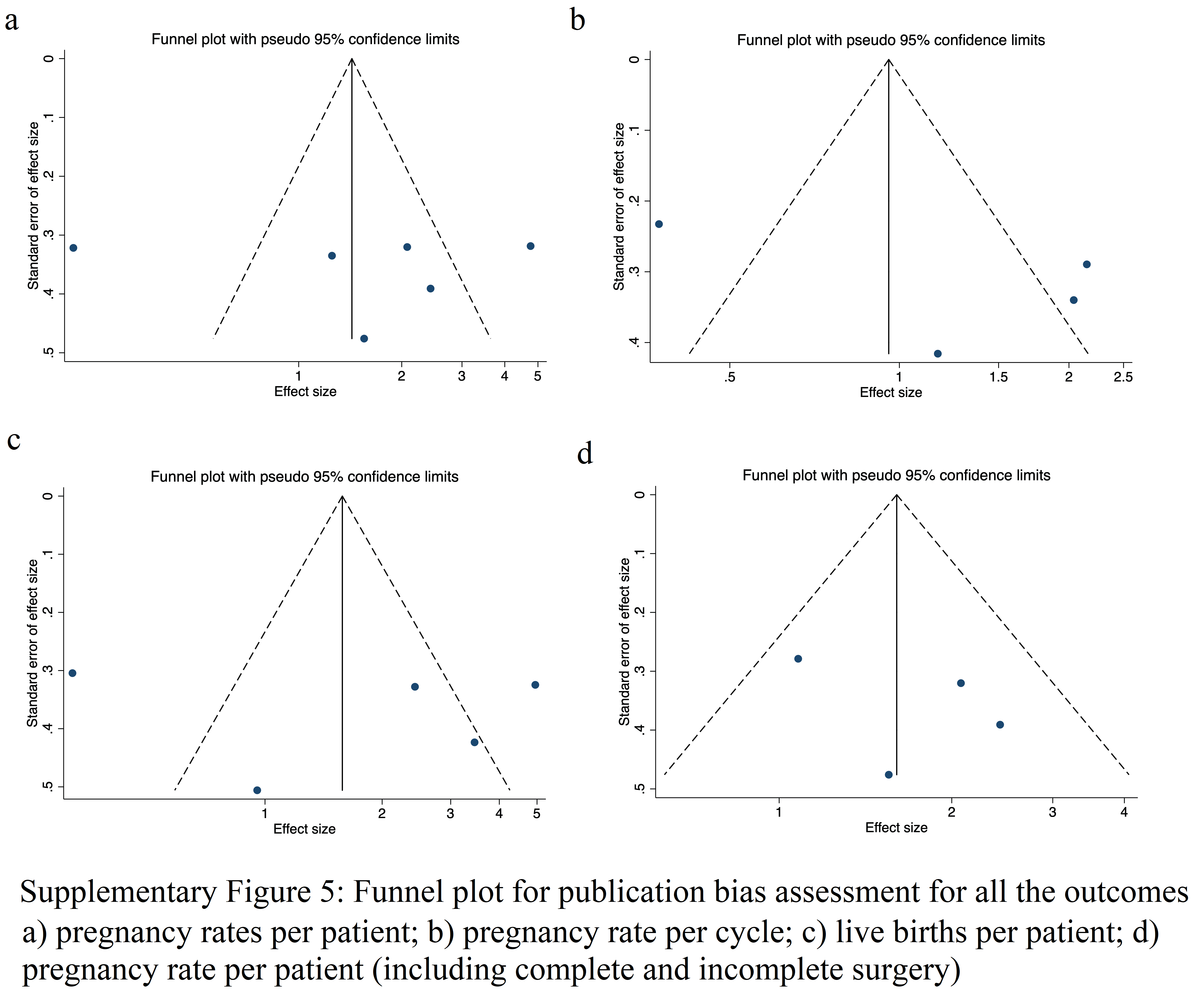

Supplement: Supplementary file 5 [file Image_5.tif]
